# Interleukin-6 regulates anti-arthritic effect of methotrexate via reduction of SLC19A1 expression in a mouse arthritis model

**DOI:** 10.1186/ar3821

**Published:** 2012-04-30

**Authors:** Misato Hashizume, Hiroto Yoshida, Keisuke Tanaka, Miho Suzuki, Isao Matsumoto, Takayuki Sumida, Masahiko Mihara

**Affiliations:** 1 Product Research Department, Fuji-Gotemba Research Laboratories, Chugai Pharmaceutical Co., Ltd., 1-135 Komakado, Gotemba, Shizuoka, 412-8513, Japan; 2 Division of Clinical Immunology, Doctoral Program in Clinical Science, Graduate School of Comprehensive Human Sciences, University of Tsukuba, 1-1-1 Tennodai, Tsukuba, Ibaraki, 305-8575, Japan

## Abstract

**Introduction:**

Methotrexate (MTX) enters cells via the reduced folate carrier SLC19A1, suggesting that SLC19A1 is associated with the efficacy of MTX. We here examined the relationship between the efficacy of MTX and the expression of SLC19A1 in glucose 6-phosphate isomerase (GPI)-induced arthritis. We found that interleukin-6 (IL-6) regulated the expression of SLC19A1, so we studied the effect of a combination of MTX and anti-mouse IL-6 receptor antibody (MR16-1).

**Methods:**

GPI-induced arthritis was induced by intradermal immunization with recombinant GPI. MTX was given from the first day of immunization. Mice were injected once with MR16-1 10 days after immunization. The levels of SLC19A1 mRNA in whole hind limbs and immune cells were measured. Synovial cells from arthritic mice were cultured with cytokines, and cell proliferation and gene expressions were measured.

**Results:**

MTX inhibited the development of GPI-induced arthritis; however, the efficacy of MTX gradually diminished. SLC19A1 expression in immunized mice with arthritis was lower than in intact mice; moreover, SLC19A1 expression in arthritic mice was further decreased when they were treated with MTX. IL-6 was highly expressed in whole hind limbs of arthritic mice. In an *in vitro *study using synovial cells from arthritic mice, IL-6 + soluble IL-6 receptor (sIL-6R) weakened the anti-proliferative effect of MTX and reduced SLC19A1 expression. Finally, although MR16-1 did not improve arthritis at all when administered on day 10, MTX in combination with MR16-1 more potently reduced the development of arthritis than did MTX alone. When used in combination with MTX, MR16-1 apparently reversed the decrease in SLC19A1 induced by MTX alone.

**Conclusions:**

In the present study, we demonstrated for the first time that IL-6 reduced the efficacy of MTX by decreasing the expression of SLC19A1, which is important for MTX uptake into cells.

## Introduction

Methotrexate (MTX) is an anchor drug for the treatment of rheumatoid arthritis (RA) because of its efficacy, acceptable safety, and cost. MTX is used in monotherapy or in combination with either biological agents or other small molecule anti-rheumatic drugs [1-3]. Regarding its anti-rheumatic mechanisms, it has been reported that MTX promotes adenosine release, inhibits pro-inflammatory cytokine production, suppresses lymphocyte proliferation, and reduces serum immunoglobulin via the inhibition of folic acid metabolism [4-6]. However, loss or reduction of its efficacy is a major problem in the treatment of RA. The efficacy of MTX varies among treated patients, and approximately 30% of patients discontinue administration within one year [7-9].

Transporters play important roles in drug disposition through their involvement in the pathways of drug absorption, distribution, and excretion, and would be among the major determinants of the pharmacological and/or toxicological effects of drugs. The ubiquitously expressed reduced folate carrier SLC19A1 is considered the major transport route for MTX [10,11]. As MTX cannot pass through the plasma membrane because of the anionic nature of MTX, SLC19A1-mediated cellular uptake should be regarded as the first step in the mode of action of MTX [12-14]. Previous studies using malignant cells showed that resistance to MTX is associated with reduced expression and activity of SLC19A1 [15,16]. However, the relationship between the efficacy of MTX and the expression of SLC19A1 in arthritic animals and RA patients is not fully understood.

Glucose 6-phosphate isomerase (GPI)-induced arthritis is widely studied, not only for the understanding of the pathogenesis of RA, but also for the development of new therapeutics, because its pathological features are similar to those of RA with pannus formation, cartilage or bone erosions, and angiogenesis in the synovium [17]. Moreover, it has been reported that cytotoxic T-lymphocyte antigen 4 immunoglobulin fusion protein (CTLA-4Ig) and antibodies to tumor necrosis factor- α (TNF-α) and IL-6, which are very effective in the treatment of RA patients [18-20], also show therapeutic effects in GPI-induced arthritis [21]. However, the efficacy of MTX has not yet been evaluated in this model.

In the present study we examined the relationship between the efficacy of MTX and the expression of SLC19A1 in GPI-induced arthritis. We found that IL-6 regulated the expression of SLC19A1, so we also studied the effect of concomitant use of MTX and anti-IL-6 receptor (IL-6R) antibody in this arthritis model.

## Materials and methods

### Animals

Male DBA/1J mice were purchased from Charles River Japan (Yokohama, Japan). The mice were specific pathogen-free and were kept in cages in a room maintained at 20 to 26°C at a relative humidity of 35 to 75%. The experimental protocol was approved by the Institutional Animal Care and Use Committee of Chugai Pharmaceutical Co., Ltd.

### Induction of glucose-6-phosphate isomerase-induced arthritis

GPI-induced arthritis was induced as previously described, with modifications [21]. In brief, male DBA/1J mice (6 weeks old) were immunized intradermally at the base of the tail with 300 μg of recombinant GPI-glutathione S-transferase fusion protein (GPI-GST) emulsified with an equal volume of complete adjuvant H37Ra (DIFCO, Detroit, MI, USA). Pertussis toxin (200 ng/200 μL) was injected on the day of immunization and 2 days after immunization. Clinical symptoms of arthritis were evaluated visually and assigned a scale of 0 to 3 for each limb (maximum score/mouse = 12).

### Treatment regimen

MTX (Sigma Aldrich, St Louis, MO, USA) dissolved in 7% sodium bicarbonate solution was given orally 3 times a week from the first day of immunization. Ten days after immunization, mice were intraperitoneally injected once with 4 mg of rat anti-mouse IL-6R monoclonal antibody, MR16-1 [22]. The vehicle group mice were administered 7% sodium bicarbonate solution orally, and were intraperitoneally injected with phosphate buffer saline (PBS). The MTX group mice were administered MTX orally, and were intraperitoneally injected with PBS. The MR16-1 group mice were administered 7% sodium bicarbonate solution orally and were intraperitoneally injected with MR16-1. The MTX plus MR16-1 group mice were administered MTX orally and were intraperitoneally injected with MR16-1. Each group consisted of 8 or 9 animals.

### Analysis of gene expressions in mouse whole hind limbs

Immediately after the animals were killed, whole hind limbs were immersed in RNAlater RNA Stabilization Reagent (Qiagen, Valencia, CA, USA) and stored at -80°C until total RNA extraction. The whole hind limbs were excised, immediately soaked in TRIzol (Invitrogen, Carlsbad, CA, USA), and crushed in a bead mill (TissueLyser II; Qiagen, Valencia, CA, USA). Total RNA was extracted using an RNeasy kit (Qiagen) according to the kit manufacturer's protocol. Amounts of total RNA obtained from a single whole hind limb were 4.5 to 18 μg (intact mice), 15to 60 μg (non-treated immunized mice), 9 to 60 μg (MTX-treated immunized mice), 12 to 30 μg (MR16-1-treated immunized mice), and 4.2 and 30 μg (MTX- and MR16-1-treated immunized mice). cDNA was synthesized with an Omniscript RT kit (Qiagen) using random 9-mer primers (TaKaRa, Shiga, Japan) according to the kit manufacturer's protocol. Quantitative real-time polymerase chain reaction (PCR) was performed by running a TaqMan gene expression assay (Applied Biosystems, Foster City, CA, USA), targeting mouse *SLC19A1, IL-6, TNF-α*, and *glyceraldehyde-3-phosphate dehydrogenase (GAPDH)*, on an ABI PRISM 7500 system (Applied Biosystems) according to the manufacturer's protocol.

### Analysis of gene expressions in mouse immune cells

For CD4 T cell and B cell subset sorting, splenocytes were labeled with antibodies to CD4 and B220 and sorted to 97% purity by using a fluorescence-activated cell sorter (FACSAria III; BD Biosciences, Franklin Lakes, NJ, USA). Total RNA was extracted using an RNeasy kit (Qiagen) according to the kit manufacturer's protocol. Synthesis of cDNA and measurement of mRNA levels by quantitative real-time PCR were performed by the same methods as described above.

### Isolation and culture of mouse synovial cells

Arthritic mice were killed and the synovial tissues removed from their hind limbs. Synovial tissues were incubated at 37°C for 180 min in α-MEM supplemented with 10% fetal bovine serum (FBS) and containing 0.5 mg/mL of Liberase Blendzyme2 (Roche Diagnostics, Basel, Switzerland). After incubation of the synovial tissues with the Liberase Blendzyme2, the resulting cells were cultured in a culture flask in α-MEM supplemented with 10% FBS and the non-adherent cells were removed and discarded. Synovial cells were then subcultured in α-MEM supplemented with 10% FBS to a density of 2 × 10^5 ^cells/35 mL in a T175 flask. In this study, synovial cells from passages two to five were used.

### Analysis of gene expressions in synovial cells

Synovial cells (5 × 10^4 ^cells/2 mL/well) were cultured in α-MEM supplemented with 10% FBS for 24 h. After this pre-culture, cells were cultured with mouse IL-6 (Peprotech, Rocky Hill, NJ, USA) and soluble mouse IL-6R (R&D Systems, Minneapolis, MN, USA), TNF-α, or MTX for 24 h. Total RNA was extracted using an RNeasy kit (Qiagen) according to the kit manufacturer's protocol. cDNA was synthesized with an Omniscript RT kit (Qiagen) using random 9-mer primers (Takara) according to the kit manufacturer's protocol. Quantitative real-time PCR was performed by running a TaqMan gene expression assay, targeting mouse *SLC19A1, multidrug resistance protein-1 (MRP-1), breast cancer resistance protein (BCRP)*, and *GAPDH*, on an ABI PRISM 7500 system (Applied Biosystems) according to the manufacturer's protocol.

### Proliferation assay

Synovial cells (5 × 10^3 ^cells/0.2 mL/well) were cultured either with medium alone or with mouse IL-6, mouse sIL-6R, and MTX in a 96-well flat bottom plate for 3 days. Synovial cell proliferation was assessed using the Cell Proliferation ELISA system (GE Healthcare UK, Buckinghamshire, UK) according to the manufacturer's instructions. Briefly, bromodeoxyuridine (BrdU) was added for the last 3 h, and its uptake was detected with anti-BrdU antibody. Substrate was added to elicit a colorimetric reaction, and absorbance at 450 nm was measured using a microplate reader (SLT Inc., Salzburg, Austria). The data are expressed in terms of optical density (OD) values.

### Measurement of the uptake of MTX

Synovial cells (5 × 10^4 ^cells/2 mL/well) were cultured for 24 h in α-MEM supplemented with 10% FBS. After this pre-culture, cells were cultured with mouse IL-6 and mouse sIL-6R for an additional 24 h. After washing cells with PBS, cells were incubated in the presence 100 nM of Alexa Fluor 488 conjugated MTX (Invitrogen) for 5 h. After incubation, cells were treated with trypsin-EDTA (Gibco, Grand Island, NY, USA) to obtain a single cell suspension. Intracellular accumulation of MTX was measured with a fluorescence-activated cell sorter (FACSCanto II; BD Biosciences) and calculated mean fluorescence intensities (MFIs) were analyzed using FACSDiva software (BD Bioscience).

### Measurement of cytokine levels in serum

Serum samples were collected 15 days after immunization, and the concentrations of serum amyloid A (SAA) and IL-6 were measured by SAA mouse ELISA kit (Invitrogen) and Mouse IL-6 Quantikine ELISA Kit (R&D systems), respectively.

### Statistical analysis

Statistical significances were estimated by Wilcoxon's test, unpaired *t*-test, and Dunnett's multiple comparison test using a statistical software package (SAS Institute Japan, Tokyo, Japan), with the significance level set to 5%.

## Results

### Efficacy of MTX, and SLC19A1 expression in GPI-induced arthritis

**Figure 1:**
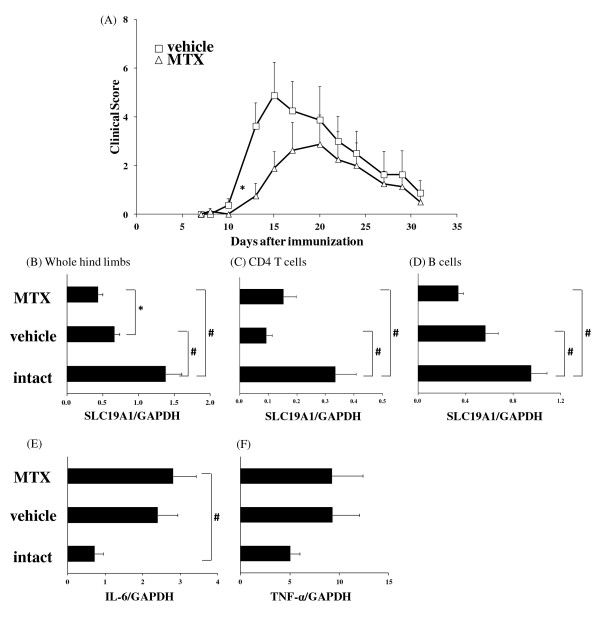
**Effect of methotrexate (MTX) in the glucose 6-phosphate isomerase (GPI)-induced arthritis model**. Mice were immunized with GPI-GST emulsified in complete adjuvant. MTX (10 mg/kg) was administered 3 times a week from the first day of immunization. (**A**) Arthritis score was assessed as described in the *Methods *section. Each symbol indicates the mean and standard error (SE) of 8 animals. Statistically significant differences in clinical scores were analyzed by Wilcoxon's test (*P < 0.05). SLC19A1 mRNA expressions in (**B**) whole hind limbs, (**C**) CD4 T cells, and (**D**) B cells were measured by real-time polymerase chain reaction (PCR). (**E**) IL-6 and (**F**) TNF-α mRNA expression in whole hind limbs was measured by real-time PCR. Samples were obtained on day 15. Each bar and error bar indicates the mean and SE of 8 animals. Statistical significance was analyzed by Dunnett's multiple comparison test (#*P *< 0.05) or unpaired *t*-test (*P < 0.05). GAPDH, glyceraldehyde-3-phosphate dehydrogenase.

MTX was administered orally 3 times a week for 4 weeks. MTX (10 mg/kg) suppressed the progression of arthritis, but from day 20 its efficacy gradually diminished (Figure 1A).

The mRNA expressions of SLC19A1 in whole hind limbs and immune cells were examined 15 days after immunization (peak of arthritis). The SLC19A1 expression in immunized mice was lower than that in intact mice (Figure 1B-D). Moreover, the expression level of SLC19A1 in whole hind limbs was further decreased by treatment with MTX (Figure 1B). TNF-α and IL-6 mRNA expressions in whole hind limbs were up-regulated in immunized mice with arthritis compared with intact mice, and MTX treatment did not reduce TNF-α or IL-6 mRNA expressions (Figure 1E, F). IL-1 mRNA expression was not detectable (data not shown).

### Effect of MTX and IL-6 on the expression of SLC19A1 in synovial cells

**Figure 2:**
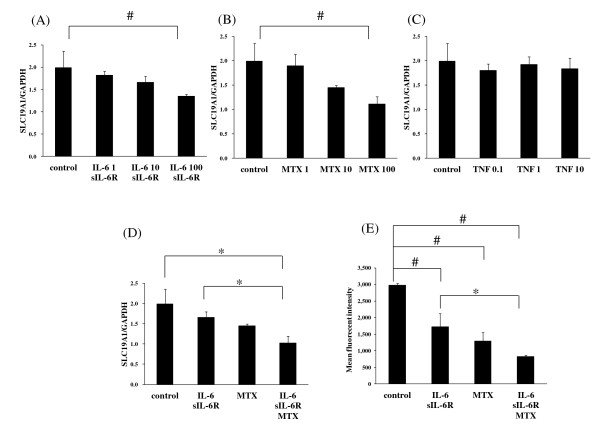
**Interleukin-6 (IL-6) and methotrexate (MTX) reduced SLC19A1 expression and uptake of MTX into mouse synoviocytes**. Synoviocytes from arthritic mice were cultured for 24 h with (**A**) IL-6 (1, 10, 100 ng/mL) + soluble IL-6 receptor (sIL-6R) (100 ng/mL), (**B**) MTX (1, 10, 100 nM), (**C**) tumor necrosis factor -α (TNF-α) (0.1, 1, 10 ng/mL), or (**D**) IL-6 (100 ng/mL) + sIL-6R (100 ng/mL), MTX (100 nM), or IL-6 (100 ng/mL) + sIL-6R (100 ng/mL) + MTX (100 nM). After culturing, cell lysate was collected and the mRNA expression for SLC19A1 was measured by real-time polymerase chain reaction. (**E**) Mouse synoviocytes were cultured for 24 h with IL-6 (100 ng/mL) + sIL-6R (100 ng/mL), MTX (100 nM), or IL-6 (100 ng/mL) + sIL-6R (100 ng/mL) + MTX (100 nM). The uptake of Alexa Fluor 488 conjugated MTX (100 nM) was quantified by measuring the fluorescence emission for each sample. Results are expressed in arbitrary units of fluorescence intensity. Each column and vertical line represents the mean and standard deviation of triplicate cultures. Statistical significance was analyzed by Dunnett's multiple comparison test (#*P *< 0.05) or unpaired *t*-test (* *P *< 0.05).

We examined the effects of MTX, TNF-α and IL-6 on the expression of SLC19A1 in synovial cells. IL-6 or sIL-6R alone did not affect SLC19A1 mRNA expression (data not shown), but IL-6 + sIL-6R significantly decreased SLC19A1 expression in synovial cells (Figure 2A). MTX, but not TNF-α, significantly decreased the expression of SLC19A1 in synovial cells (Figure 2B, C). Moreover, in the presence of IL-6 + sIL-6R, MTX further reduced SLC19A1 expression (Figure 2D).

To investigate if MTX and IL-6 + sIL-6R affected the accumulation of MTX in synovial cells, the synovial cells were pre-treated with MTX and IL-6 + sIL-6R, and then MTX uptake into cells was examined. As shown in Figure 2E, pre-treatment with IL-6 + sIL-6R, or with MTX, reduced the accumulation of fluorescent conjugated MTX in synovial cells, and pre-treatment with IL-6 + sIL-6R + MTX further reduced the accumulation of fluorescent conjugated MTX in synovial cells.

### Effect of IL-6 on MTX-induced anti-proliferative effect

**Figure 3:**
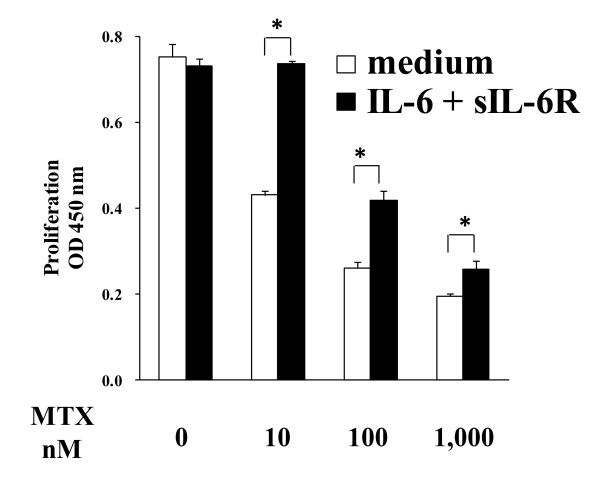
**Effect of interleukin-6 (IL-6) on methotrexate (MTX)-induced anti-proliferative effect**. Synoviocytes from arthritic mice were cultured for 24 h with methotrexate (0, 10, 100, 1,000 nM) with medium alone or in the presence of IL-6 (100 ng/mL) + soluble IL-6 receptor (sIL-6R) (100 ng/mL). After culturing, cell proliferation was measured by uptake of bromodeoxyuridine. Statistical significance was analyzed by the unpaired *t*-test (*P < 0.05). Each column and vertical line indicates the mean and standard deviation of quadruplicate cultures.

Because we previously reported that MTX suppressed the proliferation of synovial cells from RA patients [23], we examined the effect of IL-6 + sIL-6R on MTX-induced suppression of proliferation of mouse synovial cells *in vitro*. MTX clearly inhibited the proliferation of mouse synovial cells in a dose-dependent manner (Figure 3). Interestingly, the inhibitory effect of MTX was weakened by the co-addition of IL-6 + sIL-6R.

### Concomitant use of MTX and anti-IL-6R antibody in GPI-induced arthritis

Iwanami *et al. *reported that the development of GPI-induced arthritis was almost completely blocked by the injection of MR16-1 on days 0, 3, or 8 after immunization, whereas injection of MR16-1 on day 14, at the peak of arthritis, did not ameliorate arthritis, because injection of MR16-1 on day 14 did not inhibit T_h_17 induction [17]. A similar result was obtained in our study; namely, injection of MR16-1 on days 0 or 5 completely blocked the onset of arthritis, but the injection on day 10 did not ameliorate arthritis (data not shown). From these results, we decided to administer MR16-1 on day 10, because this regimen can exclude the direct effect of MR16-1 on the progression of arthritis.

**Figure 4:**
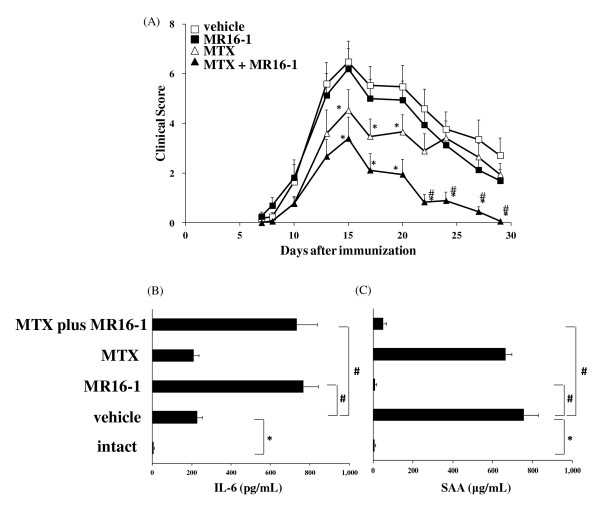
**Effect of concomitant use of methotrexate (MTX) and anti-interleukin-6 receptor (IL-6R) antibody on arthritis score**. MTX (10 mg/kg) was administered 3 times a week from the first day of immunization. Rat anti-mouse IL-6R monoclonal antibody (MR16-1) (4 mg/mouse) was injected on day 10. (**A**) Arthritis score was assessed as described in the *Methods *section. Each symbol indicates the mean and standard error (SE) of 8 to 9 animals. Statistical significance was analyzed by Wilcoxon's test (*P *< 0.05, *vs. vehicle; #*vs*. MTX). Levels of (**B**) IL-6 and (**C**) serum amyloid A (SAA) were measured by ELISA. Sera were obtained on day 15. Each bar and error bar indicates the mean and SE of 8 to 9 animals. Statistical significance was analyzed by Dunnett's multiple comparison test (#*P *< 0.05) or the unpaired *t*-test (*P < 0.05).

Next, we examined whether the combination use of MTX and MR16-1 would affect the inhibitory effect of MTX on GPI-induced arthritis. Surprisingly, although MR16-1 monotherapy did not reduce arthritis score, concomitant use of MTX and MR16-1 significantly reduced the progression of arthritis compared with the vehicle group or the MTX group (Figure 4A).

To examine whether this phenomenon was induced by blocking IL-6, we measured concentrations of IL-6 and SAA in serum on day 15. Serum concentration of IL-6 was significantly elevated in vehicle-treated arthritic mice compared with intact mice (Figure 4B). Although serum IL-6 concentration did not significantly change in the MTX group compared with the vehicle-treated group, dramatic elevation of serum IL-6 level was observed in the MR16-1 group and the MTX plus MR16-1 group. It has been shown that IL-6 induces SAA [24], and because SAA is a beneficial marker of IL-6 activity, we also measured the serum level of SAA. Levels of SAA in the vehicle group were increased to 100 times the levels in intact mice, and were only slightly reduced in MTX-treated immunized mice. On the other hand, SAA induction was completely inhibited in the MR16-1 group and the MTX plus MR16-1 group (Figure 4C). We also noted that the body weights in all groups were unchanged throughout the experiments (data not shown).

**Figure 5:**
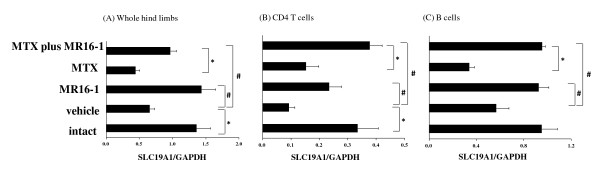
**Effect of concomitant use of methotrexate (MTX) and anti-interleukin-6 receptor (IL-6R) antibody on SLC19A1 expression**. MTX (10 mg/kg) was administered 3 times a week from the first day of immunization. Rat anti-mouse IL-6R monoclonal antibody (4 mg/mouse) was injected on day 10. SLC19A1 mRNA expression in (**A**) whole hind limbs, (**B**) CD4 T cells, and (**C**) B cells were measured by real-time polymerase chain reaction. Each bar and error bar indicates the mean and standard error of 8 to 9 animals. Statistical significance was analyzed by the unpaired *t*-test (*P < 0.05) or Dunnett's multiple comparison test (#*P *< 0.05). GAPDH, glyceraldehyde-3-phosphate dehydrogenase.

The expressions of SLC19A1 mRNA in whole hind limbs, CD4 T cells and B cells were increased in the MR16-1 group and in the MTX plus MR16-1 group compared with those in the vehicle group (Figure 5A-C). Moreover, the levels of SLC19A1 mRNA expression significantly increased in the MTX plus MR16-1 group compared with those in the MTX group.

## Discussion

Several transporters are associated with MTX uptake into cells, and these are expected to be important in determining the response and resistance to MTX [15,16]. SLC19A1 is one of the most important transporters by which MTX is taken up by cells; its expression level therefore, can predict response to MTX therapy in cancer patients [15,16]. In this study, we examined the relationship between the efficacy of MTX and the expression of SLC19A1 in an arthritic animal model. We found that: 1) the expression of SLC19A1 was significantly reduced in inflamed whole hind limbs; 2) MTX itself and IL-6 + sIL-6R, but not TNF-α, directly decreased the expression of SLC19A1 in synovial cells; 3) MTX and IL-6 + sIL-6R reduced the uptake of MTX into synovial cells and 4) the efficacy of MTX on the arthritis score was augmented by concomitant use of anti-IL-6R antibody. These results strongly suggest that the expression level of SLC19A1 is correlated with the efficacy of MTX in arthritic animals.

MTX is very effective in the therapy of patients with RA. However, the loss or reduction of its efficacy is a major problem. Although its precise mechanism is not fully understood, some reports have mentioned that specific cell membrane-associated drug efflux transporters, such as multidrug resistance protein-1 (MRP-1) and breast cancer resistance protein (BCRP), are induced upon therapy with MTX [25,26]. In the present study, we found that MTX reduced the expression of SLC19A1 in the whole hind limbs of arthritic mice. This mechanism may also be involved in secondary refractoriness to MTX in RA patients.

We also found that intracellular concentration of MTX was significantly lower in IL-6 + sIL-6R-treated synovial cells than in IL-6-nontreated cells and that the anti-proliferative effect of MTX was inhibited in the presence of IL-6 + sIL-6R. MTX enters the cells mainly via SLC19A1 and effluxes from cells via ATP-binding cassette (ABC) transporters [25,26]. As shown in Figure 2, IL-6 + sIL-6R inhibited the expression of SLC19A1. Moreover, it is reported that MTX-resistant malignant cells highly express ABC transporters such as MRP-1 and BCRP [25,26]. We examined the effect of IL-6 + sIL-6R on these two ABC transporters, but IL-6 + sIL-6R did not affect their expression (see Additional file 1). This result suggests that the decrease in intracellular concentration of MTX by IL-6 + sIL-6R results from the inhibition of MTX uptake via reduction of the influx transporter, and not by accelerated excretion of MTX from cells. MTX undergoes intracellular polyglutamation by folylpolyglutamate synthetase [27]. MTX-polyglutamates are kept within cells for longer periods than MTX itself [28]. In addition, polyglutamation increases the affinity of MTX for its target enzymes such as dihydrofolate reductase, thymidylate synthase, and 5-aminoimidazole-4-carboxamide ribonucleotide transformylase [29]. MTX-polyglutamates are converted to MTX by γ-glutamylhydrolase [30] and effluxed from cells by ABC transporters. We examined the effect of IL-6 + sIL-6R on these enzymes, but found that IL-6 + sIL-6R did not affect induction of these enzymes (data not shown).

We demonstrated that the whole hind limbs of arthritic mice showed lower SLC19A1 expression than whole hind limbs of normal mice, which was reversed by IL-6 blockade, and that MTX treatment reduced SLC19A1 expression in arthritic mice. We also obtained similar results in an *in vitro *study using synovial cells from arthritic mice; namely, IL-6 + sIL-6R and MTX each reduced SLC19A1 expression in synovial cells, and the combination of IL-6 + sIL-6R + MTX further reduced its expression. Although a precise mechanism for the reduction of SLC19A1 expression by MTX and IL-6 + sIL-6R is still unknown, our results strongly suggest that IL-6 + sIL-6R and MTX each suppressed SLC19A1 expression by an independent mechanism. We are currently planning a study to verify how IL-6 + sIL-6R and MTX suppress SLC19A1 expression.

IL-6 exerts its biological activities through two membrane molecules, a ligand-binding 80 kDa chain (IL-6R) and a non-ligand-binding signal transducer gp130 [31]. After binding of IL-6 to membrane-bound IL-6R (mIL-6R), the IL-6/IL-6R complex associates with gp130, and a signal is transmitted into the cell. In addition, sIL-6R, which lacks trans-membrane and cytoplasmic domains, can also associate with gp130 in the presence of IL-6 and transduce the signal via gp130. Therefore, both mIL-6R and sIL-6R play essential roles in IL-6 signaling. In this study, IL-6 + sIL-6R, but not IL-6 or sIL-6R alone, could decrease SLC19A1 in synovial cells, suggesting that synovial cells express gp130 but not mIL-6R. As there is sufficient sIL-6R in synovial fluid and blood, we think that the phenomenon seen *in vitro *in this study is likely to occur *in vivo*.

In RA patients as well as in arthritic animals, IL-6 concentration in serum and synovial fluid is higher than in healthy individuals or patients with osteoarthritis [32]. Therefore, IL-6-induced reduction of SLC19A1 expression is very likely to occur in patients with RA. More recently, Takeuchi *et al. *reported that combination therapy of anti-IL-6 therapy with tocilizumab and MTX showed a more remarkable effect as assessed by the Health Assessment Questionnaire-Disability Index (HAQ-DI) and by the 28-joint disease activity score (DAS28) than tocilizumab monotherapy in daily clinical practice [33]. This result could be partly explained by enhanced MTX efficacy resulting from reversal of the decrease in SLC19A1 expression by tocilizumab. Further studies of the relationship between expression of SLC19A1 and clinical response after MTX and anti-IL-6 therapy in patients with RA are needed to confirm our hypothesis.

Serum IL-6 concentration was dramatically up-regulated in the MR16-1-treated groups. We previously showed that the elevation of IL-6 levels in serum following treatment with anti-IL-6R antibodies is due to the inhibition of IL-6R-mediated clearance of IL-6 from the blood, and is not the result of induction of IL-6 protein synthesis to compensate for the IL-6 blockade or release of free IL-6 from complexes [34]. Moreover, MR16-1 treatment completely inhibited the induction of SAA. These data clearly demonstrate that IL-6 signaling was completely inhibited by MR16-1 in this study.

TNF-α antagonists in combination with MTX are highly effective treatments for severe RA [35]. When anti-TNF-α antibodies are used in monotherapy, antibodies against the anti-TNF-α antibodies are often induced. Since antibodies to the anti-TNF-α antibodies decrease serum levels of the anti-TNF-α antibody and diminish the therapeutic effects [36-39], MTX combination is mandatory in treatments with infliximab, adalimumab, or golimumab. Of interest, TNF-α-receptor-Fc-fusion protein (etanercept) has been shown to be effective for RA even as a monotherapy because antibodies against etanercept are rarely produced [40]. However, a recent report has shown that, in MTX-refractory patients with RA, clinical response is better with etanercept plus MTX than with etanercept alone [41]. Although we showed that TNF-α did not change the expression of SLC19A1, because TNF-α blockade significantly reduces serum levels of IL-6 in RA patients [42], TNF-α blockade may augment the efficacy of MTX in a manner similar to that of IL-6 blockade.

## Conclusion

In the present study, we demonstrated for the first time that the expression of the reduced folate transporter SLC19A1, which is important for MTX uptake into cells, is strongly related to the efficacy of MTX in an arthritis model. We also showed that IL-6 reduced the efficacy of MTX via the inhibition of SLC19A1 expression; therefore, IL-6 inhibition may improve responsiveness to MTX in patients with RA who show inadequate response to MTX.

## Competing interests

MH, HY, KT, MS, and MM were, or currently are, employed by Chugai Pharmaceutical., Co., Ltd.

## Authors' contributions

MH was responsible for the acquisition of data, data analysis, interpretation of data and drafting the manuscript; HY, KT, and MS for the acquisition of data; IM and TS for drafting the manuscript and MM for the interpretation of data and drafting the manuscript. All authors read and approved the final manuscript.

## Supplementary Material

Additional file 1 **A figure showing mRNA expression for multidrug resistance protein (MRP-1) and breast cancer resistance protein (BCRP)**. Synoviocytes from arthritic mice were cultured for 24 h with interleukin-6 (IL-6) (1, 10, 100 ng/mL) + soluble IL-6 receptor (sIL-6R) (100 ng/mL) or methotrexate (1, 10, 100 nM). After culturing, cell lysate was collected and mRNA expression for (A) MRP-1 and (B) BCRP was measured by real-time polymerase chain reaction. Each column and vertical line represents the mean and standard deviation of triplicate cultures. Statistical significance was analyzed by Dunnett's multiple comparison test or the unpaired *t*-test. GAPDH, glyceraldehyde-3-phosphate dehydrogenase. Click here for file
